# Ethical and Welfare Implications of Genetically Altered Non-Human Primates for Biomedical Research

**DOI:** 10.1163/25889567-BJA10002

**Published:** 2020-05-26

**Authors:** Mark J. Prescott

**Affiliations:** National Centre for the Replacement, Refinement and Reduction of Animals in Research (NC_3_Rs), Gibbs Building, 215 Euston Road, London, NW_1_ 2BE, UK

**Keywords:** animal welfare, ethics, gene editing, genetically modified, macaque, marmoset, transgenic, transgenesis

## Abstract

Breakthroughs in gene editing technologies have made it feasible to create genetically altered (GA) non-human primate (NHP) models of disease. This area of research is accelerating, particularly in China, Japan and the USA, and could lead to an increase in NHP use globally. The hope is that genetic models in animal species closely related to humans will significantly improve understanding of neurological diseases and validation of potential therapeutic interventions, for which there is a dire need. However, the creation and use of GA NHPS raises serious animal welfare and ethical issues, which are highlighted here. It represents a step change in how these highly sentient animals are used in biomedical research, because of the large numbers required, inherent wastage and the sum of the harms caused to the animals involved. There is little evidence of these important issues being addressed alongside the rapidly advancing science. We are still learning about how gene editing tools work in NHPS, and significant added scientific and medical benefit from GA NHP models has yet to be demonstrated. Together, this suggests that current regulatory and review frameworks, in some jurisdictions at least, are not adequately equipped to deal with this emerging, complex area of NHP use.

## 1 Advances in Genetic Alteration of NHPS

The ability to manipulate the genome of research animals and the explosion in the use of genetically engineered mouse and zebrafish models has enabled huge strides in understanding of biology and human disease (Vandamme, 2014; Leung & Jia, 2016). Advances in genetic technology and high evolutionary conservation of genes across vertebrates are allowing the development of new GA models in a wider range of animal species, including NHPS (Ericsson et al., 2013; Chan, 2013; Perleberg et al., 2018). Transgenic macaques were first reported over 15 years ago, and the first transgenic macaque disease model over 10 years ago (Table 1). Despite these advances, the widespread adoption of GA NHP models appeared impractical until recently, given the paucity of methods for making precise genetic changes in NHP embryos (Vermeire et al., 2017). Tools such as ZFNS (zinc finger nucleases), TALENS (transcription activatorlike effector nucleases), and CRISPR (clustered regularly interspaced short palindromic repeats, with RNA-guided nucleases such as Cas9) have made possible this once out-of-reach goal (Park & Silva, 2019). This has led to calls for the creation of lines of GA NHPS and arguments for their necessity to biomedical science (Jennings et al., 2016; Scaduto, 2016; Kishi & Okano, 2017). Germline transmission in common marmosets was first reported in 2009 (Sasaki et al., 2009), raising the possibility of establishing colonies of GA NHPS for use in biomedical research. Successful cloning of a gene-edited macaque was reported early in 2019, offering the prospect of genetically uniform gene-modified NHPS, without the disadvantages of mosaicism and cross-breeding (Liu et al., 2019).

NHPS are more similar to humans in their anatomy, physiology and behavior than are rodents. In particular, brain structures associated with higher cognition (e.g., frontal cortex) are larger and more complex in NHPS. Parts of these structures are implicated in psychiatric disorders and may have no homolog in other mammals (Wise, 2008). NHPS are therefore considered among the best animal models for complex disorders that correlate with aging, cognitive behavioral function, mental development, and psychiatric dysfunction, the societal cost of which is enormous (Izpisua Belmonte et al., 2015). It is hoped that the ability to recapitulate age-related human disease conditions in NHPS, not only physiologically but also genetically, will significantly improve understanding of the etiology of neurological and psychiatric disorders, especially those of genetic origin, and accelerate the development of effective therapies (Park & Silva, 2019). Some expect medical progress to be achieved faster than with traditional approaches such as GA rodents and non-GA NHPS. Basic research directed at understanding neural circuits of the primate brain may also benefit. For example, genetic tools such as conditional transgenesis would allow these circuits to be explored with temporal and spatial specificity, as is now routine in the mouse (Izpisua Belmonte et al., 2015).

Application of gene-editing technology to NHPS is therefore progressing rapidly, and with the increase of NHP research infrastructures in China especially, transgenic NHP models are becoming more popular. The Chinese Academy of Sciences’ Institute of Neuroscience in Shanghai has established a new 720 million yuan (US$106 million) ‘International Centre for Primate Brain Research’ with the goal to create well-established lines of cloned monkeys as animal models for brain disorders, metabolic and immune-deficiency disorders and cancer, and to make these models available to collaborating scientists around the world (Anon, 2018; Cryanoski, 2019).

The reception from the scientific community to the development of GA NHPS has been decidedly mixed. Some scientists have lauded each new advance and the potential for innovative uses. Others have questioned the utility of the new NHP models and/or suggested that existing approaches, such as GA murine models, are equally appropriate scientifically (as well as cheaper, quicker and more acceptable to the public; Cryanoski, 2019). We are still learning about how new gene editing tools act, so there is also a lot of concern about the integrity of genomic targets and efficiency. To give an example, the research team reporting autism-like behaviors in macaques engineered to carry extra copies of the MeCP_2_ gene emphasized the potential of the new model for studying autism, and that the data could not be obtained in rodent models. However, experts in MeCP_2_, Rett’s Syndrome and autism commented that symptoms observed in the macaques are less severe than those of human patients, some human MeCP_2_-duplication symptoms are absent (e.g., seizures), and expression of the gene in the macaque could be triggered by a different mechanism from that in humans, so caution should be exercised in using the model to make assumptions about human autism (Cryanoski, 2016; Dunhaime-Ross, 2016; Zeliadt, 2017; Katsnelson, 2018). At least some component of autism is human-specific and wouldn’t be seen in any animal model at all. Only time will tell whether this model can generate important, novel insights into the human condition, but clearly such scientific considerations are relevant to the question of whether it is morally defensible to genetically manipulate NHPS in this way.

Editorials in mainstream scientific journals, news items in the popular press and some review articles acknowledged ethical concerns associated with GA NHPS but rarely do they pay more than lip service to them and do not suggest how the concerns might be addressed. Meanwhile the science continues apace. In a letter responding to coverage in *Nature* of Japan’s brain-mapping project involving genetically modified marmosets, Professor Sir Patrick Bateson FRS and Dr Ian Ragan (2014) said: *“You quote US neuroscientist Terry Sejnowski, who proposes consideration of “the ethical issues that will inevitably arise up the road”. We contend that these should be considered before the journey starts”* (Bateson & Ragan, 2014: 567). Given the potential for such advances to revolutionize how NHPS are used in future research, and the high societal concern about the use of these highly sentient animals in scientific procedures generally (European Commission, 2006; Crettaz von Roten, 2012; Clemence, 2019), it is surprising there have been few focused attempts by regulatory or research funding bodies to examine the ethical issues associated with GA NHPS. Nor has there been a concerted effort to engage with the general public, which ultimately funds most of the work, to ascertain its views on the acceptability of otherwise of this new NHP research paradigm.

Two forums which have considered the ethical issues are the European Group on Ethics in Science and New Technologies (EGE) and the US National Academy of Sciences (NAS). In 2016, the European Commission tasked its Scientific Committee on Health, Environmental and Emerging Risks (SCHEER) to update an opinion on the need for NHP research and alternatives. With regard to transgenic NHPS, the Working Group, of which this author was a member, called for an *examination of “the scientific and ethical implications of such research to determine if it should be allowed in the EU and, if so, within what constraints”* (Vermeire et al., 2017: 64). The Commission passed this to EGE, an independent multidisciplinary body, but it has not yet reported on its deliberations (Moedas, 2018). In late 2018, the NAS held a workshop to explore the scientific opportunities afforded by new transgenic and chimeric NHP models in neuroscience (Bain et al., 2019), along with a related workshop on the care, use and welfare of marmosets used in gene editing-based biomedical research (Anestidou & Johnson, 2019). Both workshop reports give some attention to ethics, though not always focused on GA NHPS specifically. In this paper, I summarize the main animal welfare concerns and ethical issues, and highlight the need for appropriate oversight, taking an international perspective.

## 2 Animal Welfare Concerns

Serious animal welfare concerns arise from the generation and use of GA NHPS, which can be grouped into five broad categories—the welfare impacts of the procedures to generate the GA monkeys, the mother-infant separation, the genetic modification itself, the procedures used to study the GA monkeys, and the housing in the laboratory.

### 2.1 Welfare Impact of the Procedures to Generate the GA Monkeys

To create an animal model by way of genetic modification requires animals to undergo a number of surgical and non-surgical procedures, along with capture, handling, restraint and anaesthesia, which have the potential to cause pain and/or distress. *In vitro* production of embryos is a critical component, for either the isolation of stem cells or manipulation of the embryonic genome to create genetically modified offspring (Kropp et al., 2017). Female oocyte donors undergo repeated intramuscular injection of hormones for superovulation, and repeated blood sampling for monitoring of ovulation (Liu et al., 2014; Ramsey & Hanna, 2019). Collection of oocytes for *in vitro* fertilization (IVF) or intracytoplasmic sperm injection (icsi) is done either surgically under anesthesia or non-surgically via flushing of the uterus (Rodriguez et al., 2009; Sato et al., 2016; Niu et al., 2014). Semen is collected from males, by rectal probe electrostimulation, which requires anesthesia and carries a risk of burn injury, or by direct penile electro-stimulation whilst restrained in a primate chair (VandeVoort, 2004).

The genome is manipulated during the *in vitro* process either by microinjection of zygotes or embryos with lentiviral vectors or gene editing components using ZFNs, TALENs or CRISPR-Cas9. Manipulated embryos are then surgically implanted into surrogate dams under anesthesia (Niu et al., 2014). Analgesia is usually not mentioned in published papers, which describe the procedures in little detail. Suitable surrogates, exhibiting normal menstrual cycles, are selected based on steroid hormone profiles, requiring further blood sampling, or observation of menses. Monitoring of pregnancy and gestation is generally done using non-invasive imaging (e.g., ultrasonography), with manual or chemical restraint (Tarantal, 2005).

Mothers give birth to infants naturally or more often via caesarean section to prevent microbial infection, necessitating surgery and anesthesia. Anesthesia may be interrupted during the procedure to ensure that the neonate(s) are able to be resuscitated (Sato et al., 2016). Fertilization and pregnancy rates remain low (around 30%) and surrogates often miscarry, either as an effect of the technology or genetic alteration (see Section 3). Post-natal suffering and deaths may also be an issue. Offspring from successful pregnancies are screened for incorporation of the genetic alteration of interest, which may involve ear tissue biopsy (Liu et al., 2008).

### 2.2 Welfare Impact of Mother-Infant Separation

Surviving offspring from GA NHP programs are often nursery-reared (i.e. reared with other infants, with supplementary feeding by humans, rather than being raised naturally by their mothers), sometimes in an isolator (Chen et al., 2017; Shi et al., 2019). Images and video abound in the news outlets of infant macaques clinging to each other or fluffy toys in isolators, or showing locomotor stereotypies, seemingly without recognition by the laboratories providing the images of the ethical and welfare issues they illustrate (Zeliadt, 2017; Cryanoski, 2019). The early life rearing environment has a major influence on NHP welfare. The stress of artificial/early weaning and maternal separation can have profound consequences, leading not only to distress, fear, depression and behavioral disturbance (e.g., stereotypies), but also remarkably long-lasting detrimental changes in physiological and immunological responses, with health consequences later in life (e.g., increased vulnerability to gastrointestinal disease) (Prescott et al., 2012). These procedures are, in fact, used to create NHP models of stress, depression, grief and immune deficiency. The psychological harm, in early life and adulthood, ought to be acknowledged and factored into any harm-benefit assessments about whether and on what terms GA NHP research should proceed (see Section 5). Mothers separated from their infants show physiological and behavioral signs of stress and depression (Prescott et al., 2012).

Nursery-reared infants can lack the necessary experience to be competent mothers later in life, rejecting or mis-mothering their infants, which raises questions about the feasibility and ethical acceptability of creating selfsustaining colonies of NHPS with mutations transmitted through the germline. Another issue not addressed is the possibility that the adverse effects caused by nursery rearing may confound characterization of the phenotype and subsequent experiments, especially where nursery-reared GA animals are compared with mother-reared wild type controls. How can the researchers be sure deficiencies (e.g., in social behavior) are due to the genetic modification and not the rearing condition/environment? Some studies appear to lack controls altogether or publish few details about the founder animals. Clearly there is a need to refine and improve current practices.

### 2.3 Welfare Impact of the Genetic Modification Itself

Genetic engineering has the potential to create an array of NHP models with debilitating disease phenotypes, with Huntington’s disease, Parkinson’s disease, Duchenne muscular dystrophy and autism being some of the diseases targeted thus far. The impact on the animals’ welfare could be severe, depending on the clinical signs, humane endpoints and degree to which their locomotor, feeding, drinking, grooming, communication and other behaviors are affected. Chen et al. (2017) found their TALEN-edited MeCP_2_ mutant monkeys to exhibit more stereotypical behaviors than wild type controls (not only in frequency but also duration), in addition to social withdrawal, fragmented sleep, reduced cortical grey matter and other adverse effects expected to compromise their psychological or physical wellbeing. Cryanoski (2016: 302) writes of these animals at the Yunnan Key Laboratory of Primate Biomedical Research: *“An animal sits listless and unresponsive, holding tight to the bars of the cage as her normal twin sister crawls all over her. In another cage, a monkey with the mutation pumps its arm, reminiscent of repetitive behavior seen in the human disorder. Some incessantly suck their thumbs’”*. In the case of germline modifications, the adverse effects may be seen in all offspring and future generations.

Current gene editing techniques are not optimal, and this too could have implications for animal welfare. For example, off-targeting is a concern with the CRISPR/Cas9 system (Chen et al., 2016). Because Cas9 can induce mutations in both its on-target and off-target sites, there is the potential for unwanted or unexpected modifications (Zhang et al., 2015), which could lead to unanticipated phenotypes, deleterious side effects and/or atypical responses to housing, husbandry and experimental procedures. Thus, in practice, it is difficult to foresee the effects on modified animals accurately and to prevent unwanted suffering. This is one of the reasons why gene-editing is ethically more problematic than induction of disease states through other means, such as drugs or surgery, though variability can be an issue here too (Vitale et al., 2009).

### 2.4 Welfare Impact of the Procedures Used to Study the GA Monkeys

Phenotypic characterization of new GA monkeys is necessary and a variety of methods have been reported in the literature including MRI scanning under anesthesia, ECG recording, eye tracking, analysis of peripheral blood, pain threshold testing (hot plate), active avoidance to fear-inducing stimuli (high decibel level noise—120 dB) and other behavioral assays, all of which can compromise welfare if they cause pain, fear, discomfort, distress or other negative emotional states (Chen et al., 2017). The ultimate goal seems to be to generate cohorts of genetic disease models for the evaluation of therapeutic strategies aimed at treatment of human disease conditions. Therefore, the animal subjects can be expected to undergo administration of drugs by intended clinical routes, physiologic sampling and behavioral testing. Those used in basic neurophysiology, or modeling of brain disorders, may be expected to experience the neuroimaging, surgical implantation of recording/stimulation devices, cognitive testing, food/fluid restriction, prolonged restraint and social separation currently used in most NHP experiments in the awake, behaving state, and to be utilized over a period of many years (Prescott et al., 2010; Kishi & Okano, 2017). In the UK, such long-term protocols have a prospective severity classification of ‘severe’. This reflects the seriousness of the adverse effects or complications that can occur in a minority of animals, and the requirement under Directive 2010/63/EU for severity classification to consider the lifetime experience of animals, the duration frequency and multiplicity of harmful techniques, the potential of cumulative suffering within a procedure, and the application of refinement techniques (European Union, 2010; Pickard et al., 2013). All these animals will be eventually be killed by a permitted method. Whilst procedures in this category are not specific to genetic models, they represent additional harms that need to be factored into the overall harm-benefit analysis.

### 2.5 Welfare Impact of the Housing and Husbandry

There is a large literature on the importance for good laboratory NHP welfare of living with compatible conspecifics, housing in large, complex, enriched environments, and human-animal interactions that are based on trust and cooperation, not fear or force (Rennie & Buchanan-Smith, 2006a,b,c; Jennings & Prescott, 2009). However, provision of these crucial elements of an effective behavioral management program for NHP welfare is highly variable globally. There are many reasons for this including cultural attitudes to animals (e.g., in Eastern philosophy, preservation of animal life can be given more weight than quality of life with the consequence that experimental animals may not be euthanized after their research use has ended, even when they are kept in conditions that are evidently causing poor welfare), differing awareness of the animal welfare science and NHP management literature, and a lack of robust oversight in some cases (Prescott et al., 2017).

## 3 Number of Animals Involved

Another major ethical issue involved with GA NHP research is the large number of NHPS required to undergo regulated procedures in order to produce a handful of offspring carrying the genetic mutation that can be studied experimentally. Despite the latest technologies being described as ‘precision geneediting’ their application to NHPS remains an inefficient process. Chen et al. (2016) report that the efficiency of gene targeting with TALENS or CRISPR in NHPS ranges between 20% and 75%, depending on the targeted genes and the research group; this is more variable and lower than what is reported for mice. Some TALENS efforts have resulted in no live births. Most of the NHP founders are heterozygous chimeras and as mentioned above, off-targeting is a concern with the CRISPR/Cas9 system. The desired phenotypes do not always appear due to target genes being modified in a mosaic pattern. For ethical reasons, and in line with the reduction principle of the 3Rs (Russell & Burch, 1959; www.nc3rs.org.uk/3Rs), it is necessary to obtain the maximum number of results with as few individuals as possible. Combined with high financial/breeding costs, this may drive improvements in the precision of gene-editing technologies in the future. For example, the latest technological advance, prime editing, promises more targeting flexibility and greater editing precision but it has yet to be applied to NHPS (Anzalone et al., 2019). In a minority of cases, preimplantation diagnosis has been used to increase efficiency; for example, Sato and Sasaki (2017) selected marmoset embryos by fluorescent protein expression prior to transfer to surrogate mothers, to guarantee that all infants harbor the transgene.

**Figure 1:**
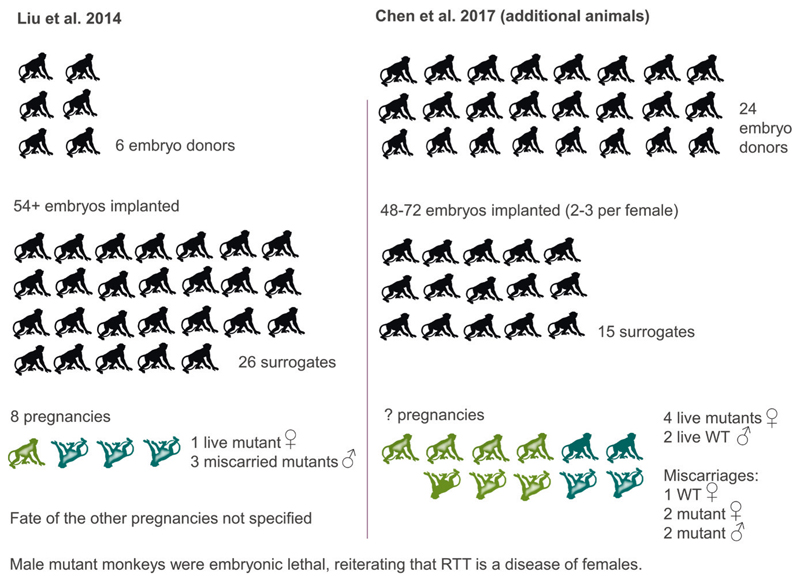
llustrative example of the large number of animals required to make GA NHP models.

The large number of animals required is best illustrated with an example (by no means the worst or best) (Fig. 1). Rett syndrome (RTT) is a rare genetic disorder that affects brain development, resulting in severe mental and physical disability. It is estimated to affect about one in 12,000 girls born each year and is only rarely seen in males. Liu et al. (2014) used a total of 32 macaques to generate one live TALEN-edited MeCP_2_ mutant monkey as model of RTT; over 54 embryos from six embryo donors were implanted into 26 surrogates, eight of which became pregnant, yielding the one live mutant female and three miscarried mutant males (male monkeys were embryonic lethal, reiterating that RTT is a disease of females); the fate of the other pregnancies was not reported. In a related, subsequent paper, Chen et al. (2017) reported additional TALEN-edited MeCP_2_ mutants. In this case, two to three embryos each were yielded from 24 embryo donors, and implanted into 15 surrogates; the number of pregnancies was not specified, but there were four live births (four mutant females and two wild-type males) and five miscarriages (two mutant males as expected, but also two mutant females and one wild-type female).

Based on these odds, to generate say six GA animals for experimental studies to investigate the effect of the transgene would require in the region of 60-190 donor and surrogate animals to undergo invasive procedures. The founders may then be used to generate additional GA animals, and historically genetic experiments use large numbers because of gene-environment interactions and genomic diversity. Now it may be that the NHP egg donors and foster mothers would not be killed but re-used, thereby helping to reduce animal use overall. This does not generally occur in GA mice programs but may be considered more important in the case of NHPS. However, this would have the effect of increasing the harm caused to those animals that are re-used (which is counter to the Refinement principle of the 3Rs). Regardless of whether reuse takes place, this example illustrates how gene editing of NHPS represents a step change in numbers and the way in which these particular animals are used in research.

This author has reviewed over 700 NHP research proposals and numerous manuscripts detailing NHP neuroscience, vaccinology, immunology and toxicology. The publication standard for neurophysiology experiments in the awake, behaving state (to understand the causal relationship between neuronal activity and cognitive function) is two macaques. Lesion, vaccine, pharmacokinetic and safety pharmacology studies generally involve around six animals. Regulatory toxicology studies of pharmaceuticals or biologics, another common use of macaques, would typically use 4–32 animals, depending on the study type and endpoints (Prior et al., 2017). In these cases, practically every animal bred/supplied is used as an experimental animal and their mothers are not required to undergo regulated procedures (with the exception of reproductive toxicology) and there is little or no wastage. Wastage of animals is something that we have become accustomed to for GA mice (though considerable efforts are made to reduce it), but it is not something that has hitherto been encountered in NHP research and therefore GA raises serious ethical issues.

It has been argued by some proponents of cloning of gene-edited NHPS, that this will reduce the number of NHPS needed for certain types of experiments, such as drug testing, by significantly reducing genetic variation between animals and hence making it easier to detect drug-induced effects with smaller sample sizes (Cryanoski, 2019). Assuming this is the case, whether it will lead to a reduction in overall NHP use for efficacy and safety studies of new drugs is not clear, especially given the high financial cost of cloning GA NHPS. The first group to do so reportedly spent half a million US dollars to create five clones from one animal, involving 325 cloned gene-edited embryos implanted into 65 surrogate monkeys (Cryanoski, 2019).

## 4 Practical and Geographical Considerations

Whether GA NHPS will replace the use of GA mice or other GA species in a major way is unknown. NHP species have long, slow life histories, small litters and much slower reproduction rates than mice (Table 2). They are expensive to purchase, transport and maintain in the laboratory, especially in environments that meet their complex behavioral, social, physiological and psychological needs. Staff members working with NHPS require specialist training (perhaps more so in the case of GA NHPS), and a high degree of PPE is necessary in some cases for health and safety. Taken together, this will put off many scientists from pursuing their research goals in GA NHPS, and NHPS are unlikely to replace mice in the near future for gene function studies (despite some terming marmosets, *“the new mouse”)*. That said, a large number of disease-relevant human genes have homologues in NHPS but not mice.

Another limiting factor on GA NHP programs is the large number of NHPS required. China has high availability of indigenous cynomolgus macaques, which are relatively cheap to purchase, labor costs are low, and the NHP research environment is less heavily regulated. This has facilitated China becoming a global center for NHP research and especially gene-editing of macaques (Cryanoski, 2019; Hao, 2007; Zimmer, 2018). The country’s 2011 five-year plan set primate disease models as a national goal, and the science ministry invested 25 million yuan (US$3.9 million) into the endeavor in 2014 (Cryanoski, 2016). In Europe there are relatively few NHP colonies and most are much smaller than those in China and the USA, so it is hard to envisage how a major genetic engineering effort could be sustained. To enter the field, some European scientists are instead collaborating with research groups overseas. However, the German Primate Center has created transgenic common marmosets carrying the GFP transgene (which has no pathogenic effect) to study and optimize reproductive biology techniques for genetic modification of this species.

In addition to their greater fecundity and faster maturity than macaque species, the small size of common marmosets (300–400 g), their relatively ease of handling and the fact they do not carry herpes-B virus, has made them attractive species for gene editing research in Japan and the USA (Schatten & Mitalipov, 2009; Kishi & Okano, 2017; Anestidou & Johnson, 2019; Cyranoski, 2019). Demand for marmosets is now so high in the USA, there is a national shortage, with some calling for sourcing of wild animals from their native Brazil (Servick, 2018). Rapid expansion of transgenic marmoset colonies is more feasible than for macaques, as germline transmission with each generation is two to three times faster (Sasaki et al., 2009). Marmosets are poised to become a prime NHP aging model, as they are short-lived and yet show age-related pathologies, including the presence of neurodegeneration, that mirror those seen in humans (Tardif et al., 2011). Use of these more primitive New World monkeys is also possibly viewed as more ethically acceptable than Old World macaque species based on the view that their small size and preference for living in family groups makes it is easier to provide for their needs in captivity (Smith & Boyd, 2002).

Few publications reporting advances in gene editing of NHPS give details of the care and welfare of the animals, instead supplying a simple statement about approval from the local Institutional Animal Care and Use Committee (IACUC). This is itself a problem, contravening the ARRIVE guidelines for high quality reporting of animal studies (Kilkenny et al., 2010). In general, standards of NHP welfare, both in legislation and practice, are lower outside of Europe, especially in terms of the quality of housing and husbandry (e.g., Chapman et al., 2015; McMillan et al., 2017; Vermeire et al., 2017). Housing in ethologically inappropriate physical and social environments constitutes another harm to NHPS’ welfare and represents an ethical conundrum for scientists seeking to collaborate with laboratories whose standards would not be acceptable under EU legislation and/or the expectations of EU funding bodies and the public (NC3Rs 2017; NC3Rs, Bbsrc et al., 2019). Whilst of value, AAALAC accreditation is no guarantee of high welfare standards and robust ethical review because it is essentially peer review with the primary reference standards used being the legislation in the host country and without detailed examination of IACUC decisions.

The Director of the Shanghai Institute of Neuroscience has stated that the International Center for Primate Brain Research will have animal care standards higher than all existing facilities in the United States and Europe (Anon, 2018), but it remains to be seen whether this is achieved in practice, especially given the current low baseline in China and the challenges involved in operating to genuine international best practice. China does not have minimum legal provisions for NHPS and its new National Standard on Laboratory Animal Welfare (which will regulate ethical review, animal welfare and administration) is not yet widely known and fully implemented (this author was involved in translating it into English: MacArthur Clark & Sun, 2020). Published literature from China calls into question whether the 3Rs are being fully applied and hence the rigor and decision making of oversight bodies such as the IACUC (see Section 5). As noted by Zhang, concerns about loose ethical standards have dogged Chinese science (Zhang, 2018). For some researchers at least, an aspiration towards high standards of care and use appears to be judged enough in terms of addressing ethical concerns associated with GA NHPS; not robust examination of whether it is morally right for the work to go ahead in the first place.

## 5 Ethical Analysis

The development and use of transgenic or GA NHPS as models of human disease raises several ethical issues. In their ethical analysis of germline transmission in marmosets, Olsson and Sandoe (2010) encapsulate the issues into one sentence: Is it ethically acceptable to take *monkeys* and *genetically modify* them in order to *develop diseases?*

### 5.1 Utilitarianism and the Harm-Benefit Assessment

In Western societies at least, the fundamental dilemma—causing animals to suffer in research aimed at alleviating or preventing human suffering or furthering scientific knowledge—is addressed using utilitarian ethics, considering the consequences of an action for all the sentient beings involved. Legislation on the protection of animals used for scientific purposes requires researchers, regulators and/or local ethics committees (e.g., IACUC, Animal Welfare Body) to assess case-by-case whether the harms caused to the animals in a proposed piece of research are outweighed by the potential benefits (usually to humans) arising from their use (Graham & Prescott, 2015). Practically all such legislation also requires the 3Rs to be applied (Russell & Burch, 1959), that is the replacement of animals with non-animal or non-sentient alternatives wherever possible, reduction of the number of animals used per experiment to the minimum consistent with the scientific objectives, and refinement of animal use and care to minimize any pain, suffering, distress or lasting harm. However, it is important to recognize that there are viewpoints other than the utilitarian one (such as that animals have intrinsic value and a right to life and freedom from suffering) and even where the 3Rs will be fully applied it does not mean that it is morally right to proceed with the proposed work.

As we have seen from Sections 2 and 3 above, gene-editing to create NHP models of disease involves very many large-brained and highly sentient animals experiencing potentially a high degree of animal suffering and/or losing their lives. From a utilitarian perspective, this can only be justified by a significant benefit for very many human patients. The difficulty is in establishing the likelihood of achieving this. Clearly, debilitating neurological and psychiatric disorders are major health issues but, as noted by the Bateson Committee which retrospectively reviewed ten years of UK publicly-funded NHP research, the size of the medical problem to which the science relates should not be accepted as sole justification for individual pieces of research (Bateson et al., 2011).

Genetic alteration of NHPS is still relatively new and while assumptions are made about the scientific value of GA NHP models over alternative approaches, their superiority in terms of translation to medical benefit has not yet been demonstrated. Such information can only be generated case-by-case, therefore making general rules difficult. As recognized by Preuss (2019), obtaining translatable results for a single gene in one species is no guarantee that other genes will yield favorable results in that species, and it is entirely possible that neither GA macaques nor GA marmosets will provide translatable results for a given gene of interest. On the other hand, it is conceivable that a GA NHP model may be generated that makes strides in terms of understanding disease mechanism and prevents the use of many more models in other species. Genetic variation in NHPS is much more extensive than variation in humans though and less well-understood. Even where GA NHPS provide unique insights and value, the time-frame to translation could be decades rather than years.

Bioethicist Carolyn Neuhaus (2017) argues that the animal welfare concerns, availability of alternative methods for studying brain disorders, and unmet expectations of benefit justify a stop to the creation of genetic NHP models to study brain disorders. Others have argued that for apes at least the moral and welfare arguments outweigh the potential benefits of any such use (Coors et al., 2010). Whilst this author does not necessarily agree with a prohibition on all GA NHP models, balancing harms and benefits in this area is exceptionally challenging for reasons summarized in Table 3.

Some GA NHP models appear to have been created because it is technologically possible rather than this being crucial for human health, and the prospect of medical benefit is entirely lacking for others. Recently, Shi et al. (2019) reported the development of transgenic rhesus monkeys to study human lineage sequences. They inserted the human gene microcephalin (MCPH1), linked to brain development, into 11 rhesus embryos via lentivirus delivery. The five surviving offspring showed extended brain development (neoteny), improved short-term memory and shorter reaction times on cognitive tests. The work was justified by the researchers as an attempt to understand the evolutionary process leading to the human brain, but it was widely condemned on ethical grounds in the international press, including by NHP researchers, with some calling the experiment “*an ethical nightmare*”. Despite this, the Chinese team has indicated its intention to go on to examine genes implicated in language development (FOXP_2_) and human intelligence (SRGAP_2_C).

What is the anticipated societal benefit from this research? Why did the local IACUC consider it to be justified? For this author, this curiosity-driven, basic research represents an example of an IACUC failing to perform a robust harmbenefit assessment and giving priority to the interests of the local researchers. Judging from the news coverage, in Western outlets at least, most would agree the work is indefensible. In this case, there is the added concern of potentially ‘humanizing’ monkeys (by inserting human genes) in a way that might lead to increased vulnerability to harm or suffering that is closer to that of humans. The more social and cognitive capabilities NHPS possess, the weightier our obligations towards them become and the more problematic it becomes to carry out invasive research upon them. The UK Academy of Medical Sciences (2011) conducted a public dialogue on animals containing human material in which the participants expressed concern about research involving the brain, especially those of NHPS, and unease concerning the transfer of human capabilities to NHPS. The Academy concluded (110): *“We recognize that research on NHPs is appropriate, and in some types of research probably essential if it is to lead to clinical benefit, but such research should remain under a high degree of regulatory scrutiny*.”

### 5.2 Are NHPS a Special Case?

Does it make a difference that these animals are NHPS? Many scientific readers will be familiar with application of the procedures listed in Section 2.1 to mice, which has become common place in biology, but their use in NHPS will be more surprising and lead to greater concern. Olsson and Sandoe considered GA marmoset disease models no more ethically problematic than murine ones (though they did not examine the animal welfare issues in detail). However, this author considers NHPS to be a special case owing to their cognitive capabilities and intense sociality (Prescott, 2010). While the biological proximity of NHPS to humans implies that they may provide more reliable models for human disorders and reactions than other animals, it also implies that their capacities and abilities are more similar to ours than those of other animals, and as a result some of the deontological considerations we have for not conducting medical experiments on non-consenting humans apply also to them (Academy of Medical Sciences, 2011). While we cannot know whether pain and distress in a mouse is the same as in monkey or human, there is a logical basis for expecting the experience in monkeys to be more similar to that of humans and for affording them a greater moral status (Boyd Group, 2002). We certainly know a great deal about the negative impacts of procedures such as social separation of NHPS, giving cause for concern. Many would agree that it is more difficult to meet the complex needs of NHPS in the laboratory environment than it is for mice, meaning that contingent suffering is also increased.

Recent years have seen major progress in Europe towards reducing and refining the use of NHPS without exporting this research overseas (Vermeire et al., 2017). There is real concern that the advent of gene editing of NHPS could reverse this decline. Will researchers seek to create thousands of NHP mutants, as occurred after the technology to genetically engineer mice first appeared? (Katsnelson, 2018). If scientists have ready access to gene-edited monkeys, might they use them for experiments that are conventionally done in rodents? (Cryanoski, 2019). Such scenarios seem more likely in some countries than others but would represent a major setback at a time when investment and activity in the 3Rs is growing. Clearly, efforts to apply the 3Rs to NHP research need to be extended to GA work.

There is greater concern among the public and scientific community about research employing NHPS compared to other animals (Aldhous et al., 1999; Clemence, 2019). Olsson and Sandoe speculated that, given this sensitivity, an increased focus on transgenic NHP research could lead to increased public resistance to animal research per se. While this might not come to pass, special concern about NHPS suggests this is an area in which we should tread very carefully. NHPS are also a special case because the ability to induce heritable changes to the germline DNA of other primate species brings us technologically closer to human genetic engineering (Schatten & Mitalipov, 2009), with all of associated ethical issues. Certainly, the hugely controversial germline gene-editing experiment of He Jiankui—to make human babies resistant to HIV—has placed NHP efforts in a more negative light and caused anxiety for NHP researchers in China.

## 6 Conclusions

It is perhaps no exaggeration to say the world is on the brink of a new age in the use of NHPS for research, with China at the vanguard. Industrial scale production of GA NHP models for export or research in countries where they are created is likely to lead to an expansion in the use of our primate cousins at a time when it is widely accepted we should be working to replace, reduce and refine their use instead. While some believe GA NHPS offer the prospect of significant advances in understanding and treatment of neurological disorders and infectious disease, this cannot be considered axiomatic and there is a danger of such models being used because of availability and novelty rather than necessity. GA technologies as applied to NHPS are still inefficient, using large numbers of animals and having unknown risks. Clearly this is an area which warrants careful ethical consideration, but evidence of this is lacking. This suggests current regulatory and review frameworks, in some jurisdictions at least, are not adequate.

Given this, the author recommends that decisions on the ethical acceptability of both the creation and use of GA NHPS should be made case-by-case at a higher level than the local IACUC or Animal Welfare Body, preferably by international organizations such as the World Health Organization and European Commission. The issues are sufficiently serious to warrant scrutiny by a higher expert oversight body. An independent committee is more likely to perform robust harm-benefit assessment, with recognition and inclusion of all known harms; realistic appraisal of the risks, probability of success and potential benefits; and consideration of alternative approaches. This would help to ensure that only projects with a very high likelihood of producing medical benefit would proceed, and that contentious research which carries a high welfare cost is adequately justified and can be defended in the public domain.

An expert oversight group could also issue guidance on when, why and how GA NHP projects should be attempted. With the right composition, knowledge and skills, this could include the areas of greatest unmet medical need for which GA NHPS are the only viable approach, what degree of harm is permissible and under what circumstances, how the 3Rs might be applied to positively shift the harm-benefit ratio, technological improvements needed to improve precision and maximize efficiency, and where the absolute limits should lie (e.g., in terms of humanizing NHPS for human benefit).

## Figures and Tables

**Table 1:** Milestones in genetic alteration of NHPS

Milestone	Species	Country	Reference
First transgenic NHP (ANDi); GFP reporter gene	*M. mulatta*	USA	Chan et al. (2001)
First transgenic NHP disease model— Huntington’s disease (HD); human mutant huntingtin gene	*M. mulatta*	USA	Yang et al. (2008)
Germline transmission (inherited vector-integrated transgene from parents); enhanced GFP gene	*C. jacchus*	Japan	Sasaki et al. (2009)
First GE NHP using TALENS—Rett syndrome (RTT); human MeCP_2_ gene	*M. mulatta* *M. fascicularis*	China	Liu et al. (2014)
First GE NHP using CRISPR/Cas9; Ppar-γ and Rag1 genes	*M. fascicularis*	China	Niu et al. (2014)
Generation of chimeric fetuses using embryonic stem cells	*M. fascicularis*	China	Chen et al. (2015)
First GE NHP using ZFNS/TALENS— model of severe combined immunodeficiency (SCID); IL2RG gene	*C. jacchus*	Japan	Sato et al. (2016)
Cloning of a gene-edited NHP using SCNT; knockout of circadian transcription factor BMAL_1_	*M. fascicularis*	China	Liu et al. (2019)

**Table 2:** Life history variables for macaques, marmosets and mice (Ash & Buchanan-Smith, 2014; Bakker et al., 2018; Gagliardi et al., 2007; Jennings & Prescott, 2009; Prescott et al., 2012)

	Rhesus macaque *Macaca mulatta*	Long-tailed macaque *Macaca fascicularis*	Common marmoset *Callithrix jacchus*	Mouse *Mus musculus*
Sexual maturity	~3 years for females, ~4 years for males	~4 years for females, ~7 years for males	~15–18 months	6 weeks for females, 8 weeks for males
Gestation time	~5.5 months	~5.5 months	~5 months	19–1 days
Typical litter size	Singleton	Singleton	Multiple offspring; most frequently twins or triplets	3–14 young
Inter-birth interval	12–24 months	12–24 months	~5 months	Typically breed for about 7–8 months, producing four or more litters
Lifespan	Average 25 years	25–30 years	Average 5–7 years	1–3 years

**Table 3:** Challenges for the harm-benefit assessment

	Benefits	Harms
Positive	– The new scientific knowledge gained may be the only way to answer questions crucial to the development of new and improved treatments for human disease. – GA NHPS might accelerate the development of effective therapies for patients.	– Genetic disease models may avoid the need for harmful induction procedures by chemical or surgical means. – If genetic models are more precise with less variability, fewer NHPS may be needed per experiment. – GA NHPS could prevent the use of models in other species.
Negative	– The public is more supportive of animal research if there is clear human benefit. As yet there is no evidence of improved translation with GA NHP models; this will only be known in the long–term. – There is no guarantee GA NHPS will be better surrogates for studying human disease than the available alternative approaches. – The desired phenotypes do not always appear, and the symptoms may not replicate the human experience. – NHPS are not always predictive of man in other fields of science. – Some studies are poorly designed and reported. Issues related to model validity could compromise translation.	– Living, sentient beings are deliberately caused to suffer disease, which we humans seek to avoid. – The NHP suffering could be severe. – It is unlikely that all sources of suffering are being considered in the harmbenefit assessment (e.g., maternal deprivation/separation). – It is not possible to accurately foresee the effects on the modified animals (unknown harms). Laboratories may be unprepared for the husbandry changes needed. – Gene editing technology is still not optimal in NHPS, so generation of GA models requires very large numbers of animals; more than are used in other fields. – Widespread use of GA NHPS (whether justified or not) could reverse the progress made in reducing NHP use. – Public concern about the use of NHPS in research is higher than for other species. – There are public sensitivities about GA, and the unnaturalness of crossing species boundaries for animals containing human material (e.g., DNA).
